# Acute high‐altitude hypoxia exposure causes neurological deficits via formaldehyde accumulation

**DOI:** 10.1111/cns.13849

**Published:** 2022-05-18

**Authors:** Xiaoyin Wang, Haochen Sun, Lili Cui, Xian Wang, Changhong Ren, Zhiqian Tong, Xunming Ji

**Affiliations:** ^1^ Department of Neurology Xuanwu Hospital of Capital Medical University Beijing China; ^2^ Capital Medical University Beijing China; ^3^ Beijing Institute for Brain Disorders Capital Medical University Beijing China; ^4^ Department of Neurology Affiliated Brain Hospital of Nanjing Medical University Nanjing China; ^5^ Beijing Key Laboratory of Hypoxia Translational Medicine, Xuanwu Hospital Capital Medical University Beijing China; ^6^ Institute of Aging, Key Laboratory of Alzheimer’s Disease of Zhejiang Province, School of Mental Health Wenzhou Medical University Wenzhou China; ^7^ Department of Neurosurgery, Xuanwu Hospital Capital Medical University Beijing China

**Keywords:** ferroptosis, formaldehyde, high‐altitude hypoxia, nano‐packed coenzyme Q10, neurological deficits

## Abstract

**Introduction:**

Acute high‐altitude hypoxia exposure causes multiple adverse neurological consequences. However, the exact mechanisms are still unclear, and there is no targeted treatment with few side effects. Excessive cerebral formaldehyde (FA) impairs numerous functions, and can be eliminated by nano‐packed coenzyme Q10 (CoQ10).

**Aims:**

In this study, we aimed to investigate whether cerebral FA was accumulated after hypobaric hypoxia exposure, and further explored the preventative effect of CoQ10 through FA elimination.

**Results:**

Accumulated cerebral FA was found in C57BL/6 mice after acute high‐altitude hypoxia exposure, which resulted in FA metabolic disturbance with the elevation of semicarbazide‐sensitive amine oxidase, and declination of aldehyde dehydrogenase‐2. Excessive FA was also found to induce neuronal ferroptosis in vivo. Excitingly, administration with CoQ10 for 3 days before acute hypobaric hypoxia reduced cerebral FA accumulation, alleviated subsequent neuronal ferroptosis, and preserved neurological functions.

**Conclusion:**

Cerebral FA accumulation mediates neurological deficits under acute hypobaric hypoxia, and CoQ10 supplementation may be a promising preventative strategy for visitors and sojourners at plateau.

## Graphical abstract

Proposed molecular mechanism of acute hypobaric hypoxia induced neurological deficits via formaldehyde accumulation. AHH, acute hypobaric hypoxia; MA, methylamine; SSAO, semicarbazide‐sensitive amine oxidase; FA, formaldehyde; ALDH2, aldehyde dehydrogenase 2; H_2_O, water; CO_2_, carbon dioxide; GSH, glutathione; GPx4, glutathione peroxidase 4; LPO, lipid peroxidation; Fe^2+^, ferrous iron.

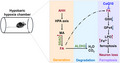

## 1 INTRODUCTION

Acute high‐altitude hypoxia exposure causes multiple adverse neurological consequences, including paralysis, blindness, sleep disturbance, mental disorder, and cognitive impairment.^1^ Intellectual abilities such as concentration, perception, executive function, learning, and memory can be impaired,^2^ which are caused by neuronal damage due to oxidative stress, inflammation, and other mechanisms.^3^ However, the exact cellular and molecular mechanism of neurological dysfunction remains unclear. Therefore, there are no targeted and effective preventions and treatments for acute mountain sickness yet.^4^

Formaldehyde (FA), a small molecular with neurotoxicity,^5^ is mainly generated by the catalysis of semicarbazide‐sensitive amine oxidase (SSAO),^6^, ^7^ and is degenerated by aldehyde dehydrogenase‐2 (ALDH2) when overproducted.^8^, ^9^ The expression and activity of SSAO are increased, while the activity of ALDH2 is decreased after ischemic‐hypoxia in mice.^10^ These changes are correlated with impaired neurological functions,^11^ and may be due to the increased FA concentration.^12^ However, the exact changes of FA and SSAO/ALDH2 under acute hypobaric hypoxia remain ambiguous. Furthermore, an in vitro experiment showed that FA exposure induced neuronal ferroptosis recently.^13^ Whether endogenous accumulated FA results in neurological decline via neuronal ferroptosis in vivo under high‐altitude hypoxia also remains to be explored.

In this study, we investigated whether endogenous FA was accumulated in the neurons after acute hypobaric hypoxia exposure via the elevation of SSAO and reduction of ALDH2, and caused subsequent neuronal ferroptosis in animal experiments. Moreover, we aimed to explore whether nano‐packed coenzyme Q10 (CoQ10), an endogenous FA scavenger,^14^ can be a potential preventive strategy for the neurological deficits under acute hypobaric hypoxia.

## 2 METHODS

### 2.1 Animal models

Male C57BL/6 mice as an animal model for acute hypobaric hypoxia studies have been well established and thus selected in this study.^15^, ^16^ Adult male C57BL/6 mice (25–30 g, 8–10 weeks, Experimental Animal Center of Capital Medical University) were housed in a temperature‐controlled room under a 12/12‐h light–dark cycle with access to water and food ad libitum. A total of 216 mice were used in this study. All experimental procedures were approved by the Institutional Animal Care and Use Committee of Capital Medical University (AEEI‐2021‐087) and the data reporting has followed the Animal Research: Reporting of In Vivo Experiments (ARRIVE) guidelines 2.0.^17^

**FIGURE 1:**
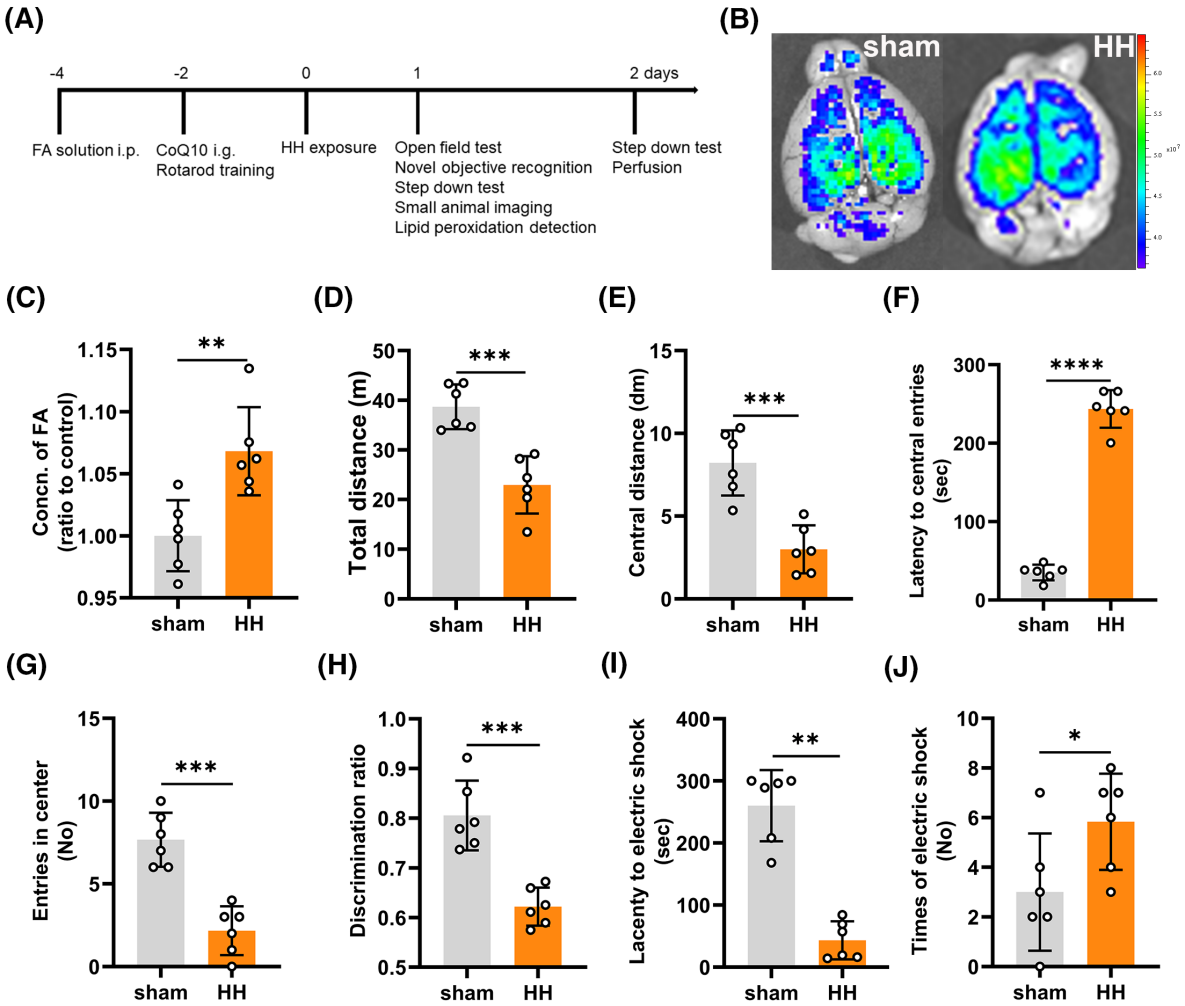
Acute hypobaric hypoxia caused formaldehyde (FA) accumulation and neurological deficits. (A) The time point of FA injection, intragastric administration of CoQ10, and hypobaric hypoxia exposure, as well as behavioral tests (D0: the hypobaric hypoxia exposure day). (B) Cerebral formaldehyde imaging using an in vivo small animal system with NaFA probe (λex/em = 440/550 nm). (C) Cerebral FA detection using QuantiChrom FA assay kit (*p* = 0.0044). (D–G) Neurological functions evaluated by open field test to show the total distance (*p* = 0.0004) (D), central distance (*p* = 0.0004) (E), latency to central entries (*p* < 0.0001) (F), and the frequency entering the center (*p* = 0.0001) (G) of each group on the first day after hypobaric hypoxia exposure. (H) Cognitive function evaluated by novel objective recognition (*p* = 0.0002) on the same day as open field test. Learning and memory ability assessed by step down test and showed the latency to electric shock (*p* = 0.0022) (I), and times of electric shock (*p* = 0.0468) (J) of each group on the second day after hypobaric hypoxia exposure. FA: formaldehyde; CoQ10: nano‐packed coenzyme Q10; sham: unexposed control group; HH: acute hypobaric hypoxia group. *N* = 6 in each group, with three independent experiments. Data are presented as mean ± SD; *p < 0.05, **p < 0.01, ***p < 0.001, ****p < 0.0001

The mice were randomly allocated into six groups (*n* ≥ 6 per group). Group 1: Unexposed control group (sham). Group 2: Exposed to acute hypobaric hypoxia (HH). Group 3: Intraperitoneal injection of FA solution (FA). Group 4: Unexposed to HH but intragastrically administrated with CoQ10 (CTL). Group 5: Intragastric administration with CoQ10 prior to HH exposure (HH + CoQ10). Group 6: Intraperitoneal injection of FA and intragastric administration with CoQ10 (FA + CoQ10). Simulated acute hypobaric hypoxia exposure was performed in an animal hypobaric hypoxia chamber (temperature: 25℃, humidity: 45%–55%) maintained at an altitude of 7000 m (41.043 kPa, equivalent to 7.8% O_2_ at sea level) (Fenglei, China) for 24 h.^15^, ^16^ FA solution was injected intraperitoneally for continuous 5 days (0.5 mM, 0.5 ml, once a day). The micellized CoQ10 was administrated orally for continuous 3 days (200 mg/kg body weight of mice, 0.5 ml, once a day), which matches with the use in clinic. The mice in the control group were maintained in the normobaric normoxic condition within the same room. The time points of FA injection, CoQ10 administration and HH exposure were shown in Figure 1A. Fortunately, there was no accidental death of mice during the experiments.

### 2.2 Behavioral tests

Open field test, novel objective recognition, rotarod test and step down test were performed to investigate the neurological dysfunction after 24 h hypobaric hypoxia exposure or 5 days FA injection. The time points of behavioral tests were illustrated in Figure 1A.

Open field test was performed to evaluate the global neurological function of the mice, especially anxiety. Mice were placed in a square of 50 cm × 50 cm × 50 cm polyvinyl chloride (PVC) box with a camera monitoring the movement into and around the central and peripheral areas for continuous 10 min’ free activity. The distance travelled in the total area and center zone, and the latency or entries into the center zone were analyzed to assess the locomotion and anxiety of the mice.

Recognition memory was analyzed by novel objective recognition.^18^ Briefly, after acclimatization, animals were placed into the PVC box with two familiarized objects for 15 min’ spontaneous activity. One hour later, the mice were placed back to the apparatus with one familiarized object and one novel object (the same position as before) for 5 min’ recognition. A discrimination ratio (novel object interaction/total interaction with both objects) was measured to assess the learning and memory of the mice.

Rotarod test was applied to test the locomotion of mice. Animals were trained for three consecutive days (three trials everyday) before test, with an accelerating rotational speed (from 4 to 40 rpm in 5 min) for 5 min. After HH and FA exposure, the mice were tested in the same patten as training. The latency to the first fall off the rod or passive movement was recorded to assess the motor function of the mice, especially coordination and athletic endurance.

As to step down test, animals were acclimated in the cage for 5 min with the current off, and then 5 min with the current on. On the second day, the animals were tested one by one for 5 min. The latency to electric shock and the times of electric shock were analyzed to assess the cognition of the mice.

### 2.3 Immunofluorescent staining

Microtubule‐associated protein 2 (MAP2) and myelin basic protein (MBP) staining were used to assess the cerebral structural damage of the mice, including prefrontal cortex (PFC), hippocampus (HPC), striatum, amygdala, and corpus callosum (CC). MAP2, synaptophysin (SYP), and postsynaptic density 95 (PSD‐95) were used to assess the injuries of in vitro cultured neurons. After acute HH and exogenous FA exposure, the mice were anesthetized and intracardially perfused with cold saline and 4% paraformaldehyde. The brain was then removed, fixed with 4% paraformaldehyde for 24 h, and embedded with optimal cutting temperature (O.C.T.) compound in liquid nitrogen. Coronal sections of 30 μm thickness were prepared. Sections (or neurons) were washed with phosphate buffered saline (PBS) followed by 0.3% Triton X‐100/PBS, blocked with 1% bovine serum albumin (BSA) at room temperature for 1 h, incubated with rabbit anti‐MAP2 (1:500, Abcam, ab32454) and rabbit anti‐MBP (1:500, Abcam, ab218011) [in vitro experiments: rabbit anti‐MAP2 (1:500), rabbit anti‐SYP (1:250, Abcam, ab52636), and rabbit anti‐PSD‐95 (1:500, GeneTex, GTX133167)] overnight at 4℃ followed by secondary antibody (goat anti‐rabbit IgG (H+L) cross‐absorbed secondary antibody, Alexa Fluor 488, 1:1000, Invitrogen, A‐11008) at room temperature for 2 h. Sections were mounted using antifade mounting medium with 2‐(4‐Amidinophenyl)‐6‐indolecarbamidine dihydrochloride (DAPI) (Vector, H‐1200). All images were taken with laser scanning confocal microscope (Zeiss LSM 880) and analyzed by WCIF ImageJ software.

### 2.4 Cerebral formaldehyde imaging

A free FA fluorescence probe NaFA was used to determine the cerebral FA levels after acute hypobaric hypoxia. The mouse brain was taken out 30 min after intraperitoneal injection of NaFA probe (10 µM, 0.5 ml),^19^ and then used for animal imaging by a small animal imaging system (Fx Pro, Carestream Health).

### 2.5 Measurement of FA, SSAO, ALDH2, iron, GSH, GSSG, GPx4, and MDA

The concentration of FA (Sigma, MAK131) in the brain or primary neurons, the activity and expression of SSAO (GENMED, GMS50538.2; Meilian, JK023695) and ALDH2 (GENMED, GMS50300.2; Meilian, JK052124), as well as the concentrations of iron (Solarbio, BC4355), Glutathione (GSH) and oxidized glutathione (GSSH) (Beyotime, S0053), glutathione peroxidase 4 (GPx4) (Meilian, MM‐44846M1), and malondialdehyde (MDA) (Beyotime, BC0025) in the cerebrum were detected using commercially available kits following the manufacturers’ instructions.

### 2.6 Lipid peroxidation detection

The mouse cerebrum was removed and incubated for 30 min at 37°C in 10 ml of PBS containing 100 U/ml collagenase IV (Solarbio, C8160) and 20 U/ml DNase I (Solarbio, D8071) after anesthetization and intracardial perfusion. Brain tissue was passed through a tissue grinder and cells were recovered after centrifugation at 400 *g* for 10 min at room temperature and separated from myelin and debris in 70% and 30% isotonic Percoll gradient (Solarbio, P8370). Samples were centrifuged at 1000 *g* for 30 min without acceleration or brake. Cells were collected from the interface and washed once with PBS.^20^ After sample preparing, lipid peroxidation (LPO) of the cerebrum was detected by BODIPY™ 581/591 C11 (Invitrogen, D3861) according to the manufacture's instruction. Cells were incubated at 4℃ for 30 min with the LPO sensor and washed with PBS for three times at 1000 *g* for 5 min. Data acquisition was carried out in a BD FacsCantoII cytometer (BD Biosciences) using the FacsDiva software (BD Biosciences) and detected at two separate wavelengths (PE for the reduced dye; FITC for the oxidized dye). The ratio of FITC/PE gives the read‐out for LPO in cells.

### 2.7 Culture of mouse Neuro‐2a cells and primary neurons

The mouse Neuro‐2a cells (N2a, ATCC^®^ CCL131™) were cultured in Dulbecco's Modified Eagle's Medium (DMEM, Gibco, 2311542) with 10% fetal bovine serum (FBS, Gibco, 10099141) and 1% penicillin/streptomycin (PS, Gibco, 15070063). Primary neurons were prepared from 18‐days old embryos as described previously.^21^ Briefly, freshly dissected cerebrums were dissociated and the neurons were seeded at a density of 9 × 10^5^ cells/ml on poly‐d‐lysine‐coated (ST508, Beyotime) dishes in neurobasal medium (Gibco, 21103049) with 2% B27 (Gibco, 17504044), 1% Glutmax (Gibco, 35050079), and 1% PS. Cytarabine (10 μM/L) (Sigma, PHR1787) was applied at 48 h to kill the glia. Upon maturation (usually about 7 days), the neurons of the hypoxic group were cultured in 1% O_2_ ^15^ (calculated with alveolar gas equation according to the altitude of 7000 m and the capillary blood flow rate and metabolism.^22^, ^23^ The normal concentration of oxygen in the brain is about 4.4%^24^), 5% CO_2_ at 37℃ in the incubator (Smartor118/118pro) for 4, 8, and 12 h, respectively. The concentration of FA incubation was 0.5 mM (in completed cultural medium) and last for 8 h. Neurons were incubated in CoQ10 (1 mM, in PBS) for 30 min before detection.

### 2.8 Detection of cytoplasmic formaldehyde

FA concentrations in the cytoplasm of these neurons were determined using a NaFA probe as described previously.^19^ In brief, to quantify cytoplasmic FA concentrations by using NaFA probe in these cultured cells, the culture medium of the cells was changed to a fresh media with 5 μM probe, and then incubated for 30 min. Subsequently, the medium was removed and washed three times with PBS to remove the excess probe. The changes of FA concentrations in these cells were quantified using a confocal laser scanning microscope (Zeiss LSM 880).

### 2.9 Statistical analysis

Statistical analysis was performed using GraphPad Prism. Kolmogorov–Smirnov test was applied to check the normal distribution of the data, while *F*‐test was used to check homogeneity of variances. Data were presented as mean ± standard deviation (SD), and were compared between groups using two‐tailed *t*‐test or One‐way ANOVA when normally distributed. Mann–Whitney *U* and Kruskal–Wilcoxon tests were performed if the data were not normally distributed. *p* < 0.05 was considered statistically significant.

## 3 RESULTS

### 3.1 Acute high‐altitude hypoxia induced cerebral formaldehyde accumulation and neurological deficits

Using a small animal imaging system with the free FA fluorescence probe NaFA (λ_ex/em_ = 440/550 nm), we found that acute hypobaric hypoxia induced a significant elevation of fluorescence intensity due to cerebral FA accumulation compared to the sham group (Figure 1B). This was further confirmed using the QuantiChrom FA assay kit (*p* = 0.0044) (Figure 1C).

In the open field test (trail diagram shown in Figure S1), the total distance (*p* = 0.0004) (Figure 1D) and central distance (*p* = 0.0004) (Figure 1E) of the HH group was significantly decreased than that of the sham ones. In addition, the latency to the central zone was significantly increased (*p* < 0.0001) (Figure 1F), while the frequency entering the center were significantly decreased in the HH group than that of the sham group (*p* = 0.0001) (Figure 1G). These results indicated that acute hypobaric hypoxia induced anxiety of the mice. Though the total distance was decreased, the results of rotarod test showed that the locomotive and coordinative ability were not significantly impaired after acute hypobaric hypoxia (*p* > 0.9999) (Figure S2). Furthermore, the results of novel objective recognition showed that the discrimination ratio was decreased significantly than that of the sham group (*p* = 0.0002) (Figure 1H). Meanwhile, the latency to electric shock was shorter after acute hypobaric hypoxic exposure (*p* = 0.0022) (Figure 1I), while the time to be shocked was increased significantly (*p* = 0.0468) (Figure 1J) than the sham ones, indicating that acute hypobaric hypoxic exposure also impaired the ability of learning and memory of the mice.

### 3.2 Acute high‐altitude hypoxia induced formaldehyde accumulation by disturbing its metabolism

**FIGURE 2:**
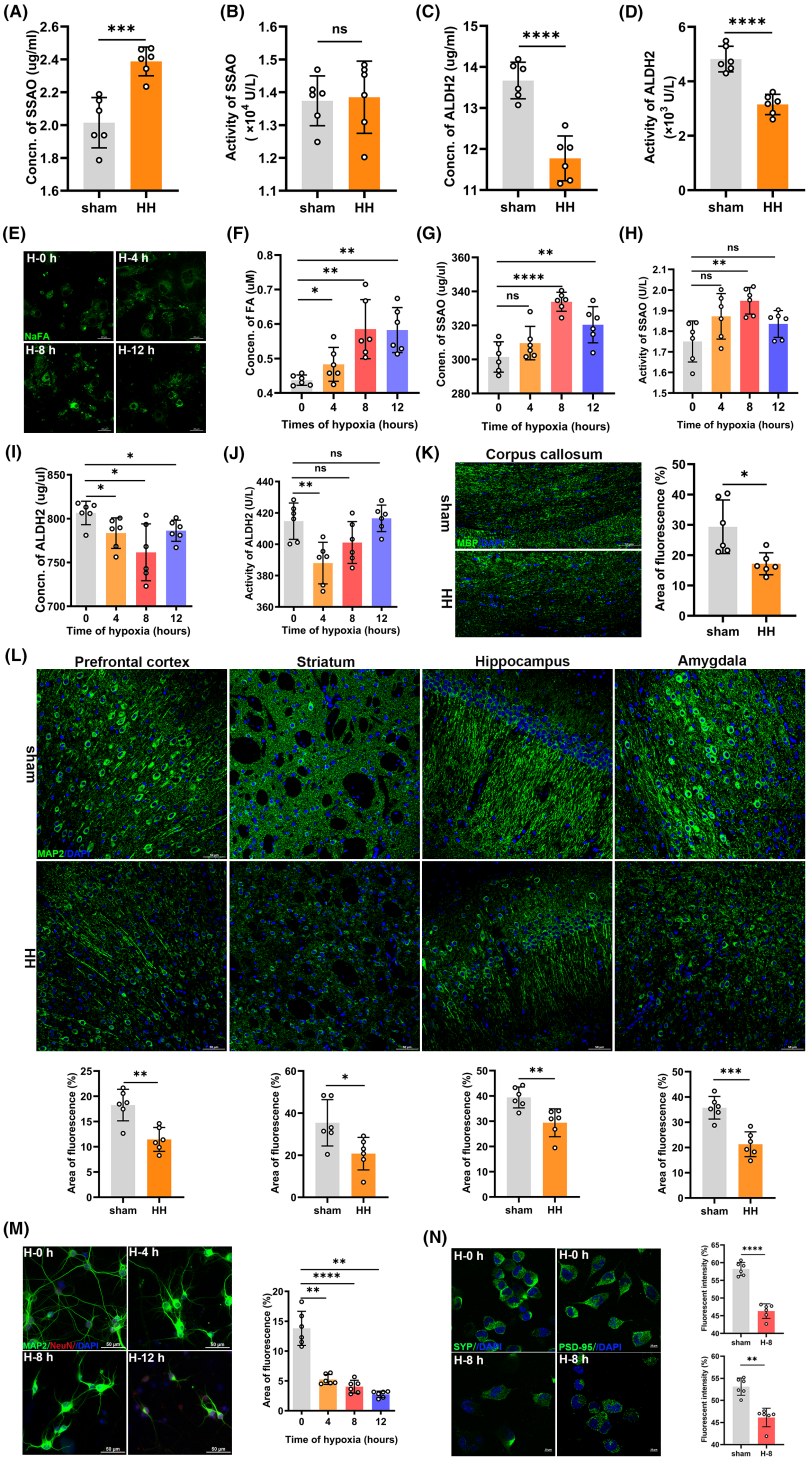
Acute hypobaric hypoxia induced formaldehyde (FA) accumulation by disturbing FA metabolism in vivo and in vitro, and induced subsequent neuronal injuries. Change of the expression (*p* = 0.0004) (A) and activity (*p* = 0.8455) (B) of SSAO in the cerebrum of the mice under acute hypobaric hypoxia. Change in the level (*p* < 0.0001) (C) and activity (*p* < 0.0001) (D) of ALDH2 in the cerebrum of the mice after acute hypobaric hypoxia. (E) Cytoplasmic FA of primary neurons after 0, 4, 8, and 12 h hypoxia detected by confocal imaging with NaFA. Scale bar = 20 μm. (F) Neuronal FA detected by QuantiChrom FA assay kit (H‐0 h vs. H‐4 h, *p* = 0.0260; H‐0 h vs. H‐8 h, *p* = 0.0022; H‐0 h vs. H‐12 h, *p* = 0.0022). Changes of the expressions (H‐0 h vs. H‐8 h, *p* < 0.0001; H‐0 h vs. H‐12 h, *p* = 0.0075) (G) and activities (H‐0 h vs. H‐8 h, *p* = 0.0022; H‐0 h vs. H‐12 h, *p* = 0.1102) (H) of SSAO in the primary neurons after hypoxia. Changes of the concentrations (H‐0 h vs. H‐4 h, *p* = 0.0279; H‐0 h vs. H‐8 h, *p* = 0.0105; H‐0 h vs. H‐12 h, *p* = 0.0201) (I) and activities (H‐0 h vs. H‐4 h, *p* = 0.004) (J) of ALDH2 in the primary neurons after hypoxia. (K) Changes of the white matter injury of corpus callosum detected by MBP (green), DAPI (blue, a dye for nuclear), using confocal imaging (*p* = 0.0107). Scale bar = 50 μm. (L) Changes of the neuronal injuries in prefrontal cortex (*p* = 0.0017), striatum (*p* = 0.0235), hippocampus (*p* = 0.0052), and amygdala (*p* = 0.0003) detected by MAP2 (green) using confocal imaging. Scale bar = 50 μm. (M) Changes of primary neurons quantified by area of fluorescence of MAP2 (H‐0 h vs. H‐4 h, *p* = 0.0022; H‐0 h vs. H‐8 h, *p* < 0.0001; H‐0 h vs. H‐12 h, *p* = 0.0022). Scale bar = 20 μm. (N) Changes of presynaptic protein (H‐0 h vs. H‐8 h, *p* < 0.0001) and postsynaptic protein (H‐0 h vs. H‐8 h, *p* = 0.0022) quantified by fluorescent intensity of SYP and PSD‐95 respectively. Scale bar = 20 μm. FA, formaldehyde; sham, unexposed control group; HH, acute hypobaric hypoxic group; SSAO, semicarbazide‐sensitive amine oxidase; ALDH2, aldehyde dehydrogenase 2. H‐0 h, hypoxia for 0 h; H‐4 h, hypoxia for 4 h; H‐8 h, hypoxia for 8 h; H‐12 h, hypoxia for 12 h; MBP, myelin basic protein; MAP2, microtubule‐associated protein 2; DAPI, 2‐(4‐Amidinophenyl)‐6‐indolecarbamidine dihydrochloride; SYP, synaptophysin; PSD‐95, postsynaptic density 95. *N* = 6 in each group, with three independent experiments. Data are presented as mean ± SD. ns: *p* > 0.05; *p < 0.05, **p <0.01, ***p < 0.001, ****p < 0.0001

To investigate the molecular mechanism of FA accumulation, we analyzed the expression and activity of SSAO and ALDH2, respectively. Our results showed that the expression of SSAO was markedly elevated in the HH group than that in the sham group (*p* = 0.0004) (Figure 2A), whereas the activity of SSAO exhibited a slight change under acute hypobaric hypoxia (*p* = 0.8455) (Figure 2B). Meanwhile, both the concentration (*p* < 0.0001) (Figure 2C) and activity (*p* < 0.0001) (Figure 2D) of ALDH2 in the HH group declined significantly compared with the sham group. Therefore, both the activation of SSAO and inhibition of ALDH2 led to the accumulation of FA in the brain of the mice under acute high‐altitude hypoxia.

### 3.3 Acute hypoxia induced formaldehyde accumulation in the primary neurons

Next, we investigated whether acute hypoxia induced FA accumulation in the cultured primary neurons. After culturing the primary neurons in a hypoxic incubator for 4, 8, and 12 h, respectively, we found the FA rapidly generated in the cytoplasm of the neurons as detected by the NaFA probe (Figure 2E) and the FA assay kit (H‐0 h vs. H‐4 h , *p* = 0.0260; H‐0 h vs. H‐8 h, *p* = 0.0022; H‐0 h vs. H‐12 h , *p* = 0.0022) (Figure 2F). Therefore, acute hypoxia caused the gathering of FA in the neurons.

Similarly, the expression (H‐0 h vs. H‐8 h, *p* < 0.0001) (Figure 2G) and activity (H‐0 h vs. H‐8 h, *p* = 0.0022) (Figure 2H) of SSAO were increased after acute hypoxia, especially at 8 h of hypoxia. The expression of ALDH2 was decreased gradually within 8 h of hypoxia (H‐0 h vs. H‐8 h, *p* = 0.0105) (Figure 2I), and the activity of ALDH2 was sharply decreased at 4 h of hypoxia (H‐0 h vs. H‐4 h, *p* = 0.004) (Figure 2J). Taken together, acute hypoxia indeed disturbed FA metabolism and resulted in the accumulation in the primary neurons in vitro.

### 3.4 Acute hypoxia induced neuronal injuries via formaldehyde accumulation

To explore whether neurological deficits resulted from the neuronal injuries after acute hypoxia, we conducted animal and cellular experiments with immunofluorescent staining. We observed multiple injuries in different brain regions (Figure S3) of the mice under acute hypobaric hypoxia compared to the sham group, including corpus callosum (*p* = 0.0107) (Figure 2K), prefrontal cortex (*p* = 0.0017), striatum (*p* = 0.0235), hippocampus (*p* = 0.0052), and amygdala (*p* = 0.0003) (Figure 2L). Consistently, in vitro experiments also revealed the loss of primary neurons under hypoxic conditions (H‐0 h vs. H‐8 h, *p* < 0.0001) (Figure 2M). The decreased expression of presynaptic (*p* < 0.0001) and postsynaptic proteins (*p* = 0.0022) indicated the reduction of the synaptic connections of the neurons after 8 h hypoxia (Figure 2N). These data suggested the neuronal damage under acute high‐altitude hypoxia, possibly mediated by FA accumulation, contributing to the neurological dysfunction.

### 3.5 Nano‐packed coenzyme Q10 prevented formaldehyde accumulation in the cerebrum

**FIGURE 3:**
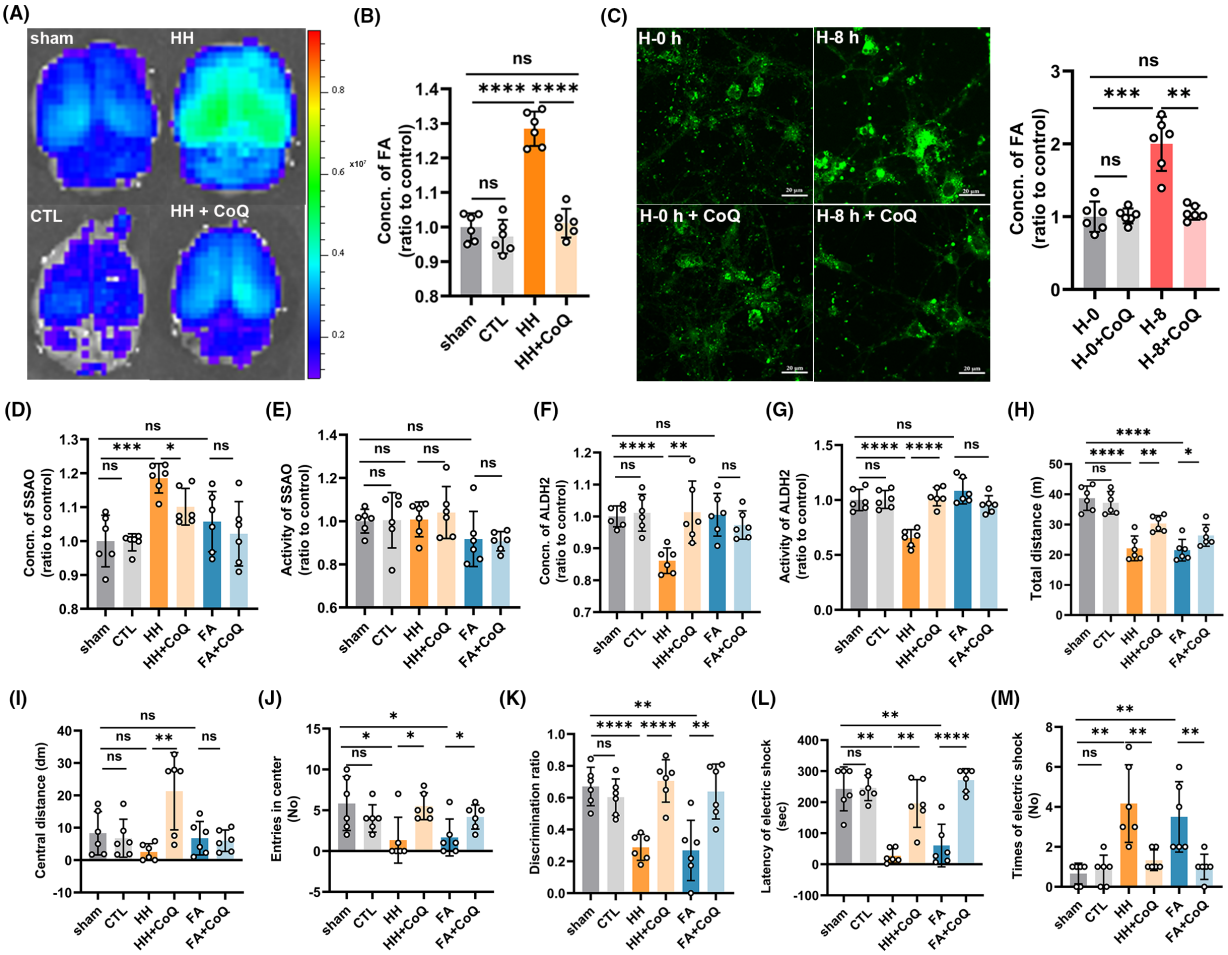
Nano‐packed coenzyme Q10 prevented formaldehyde (FA) accumulation induced by acute hypoxia and alleviated neurological deficits. (A) Cerebral FA imaging by an in vivo small animal system with NaFA probe (λex/em = 440/550 nm). (B) Cerebral FA detected by QuantiChrom FA assay kit (HH vs. HH + CoQ10, *p* < 0.0001). (C) Cytoplasmic FA of primary neurons detected by confocal imaging with NaFA (H‐8 h vs. H‐8 h + CoQ10, *p* = 0.0022). Scale bar = 20 μm. Change of the expression (HH vs. HH + CoQ10, *p* = 0.0141) (D) and activity (HH vs. HH + CoQ10, *p* = 0.5990) (E) of SSAO in the cerebrum of the mice after intervention. Change in the level (HH vs. HH + CoQ10, *p* = 0.0054) (F) and activity (HH vs. HH + CoQ10, *p* < 0.0001) (G) of ALDH2 in the cerebrum of the mice after intervention. (H–J) Neurological functions evaluated by open field test to show the total distance (HH vs. HH + CoQ10, *p* = 0.0020) (H), central distance (HH vs. HH + CoQ10, *p* = 0.0043) (I), and the frequency entering the center (HH vs. HH + CoQ10, *p* = 0.0325) of the mice on the first day after hypobaric hypoxia exposure (D1) (J). (K) Cognitive function evaluated by novel objective recognition (HH vs. HH + CoQ10, *p* < 0.0001) on D1. Learning and memory ability assessed by step down test shown as the latency to electric shock (HH vs. HH + CoQ10, *p* = 0.0022) (L) and times of electric shock (HH vs. HH + CoQ10, *p* = 0.0065) on the second day after hypobaric hypoxia exposure. (M). FA: formaldehyde; sham: unexposed control group; CTL: unexposed to HH but intragastrically administrated with CoQ10; HH: exposed to acute hypobaric hypoxia; HH + CoQ: intragastric administration with CoQ10 prior to HH exposure; FA + CoQ: intraperitoneal injection of FA and intragastric administration with CoQ10. *N* = 6 in each group, with three independent experiments. Data are presented as mean ± SD; ns: *p* > 0.05, *p < 0.05, **p <0.01, ***p < 0.001, ****p < 0.0001

Our previous studies have found that CoQ10 can combine with FA for elimination.^14^, ^25^ Whether administration with CoQ10 before hypoxia exposure could prevent FA accumulation is still not clear. Therefore, we treated the mice with CoQ10 for 3 days before acute hypobaric hypoxia exposure and found that the levels of FA in the cerebrum were significantly reduced (HH vs. HH + CoQ10, *p* < 0.0001) (Figure 3A,B). Meanwhile, the concentrations of FA in the neurons incubated with CoQ10 were also decreased, compared to the non‐treated ones (H‐8 h vs. H‐8 h + CoQ10, *p* = 0.0022) (Figure 3C). The in vivo and in vitro data indicated that CoQ10 can prevent FA accumulation in the neurons and in the cerebrum under acute hypoxia conditions.

To explore whether CoQ10 influences FA metabolism to prevent its accumulation, apart from combination, we evaluated the concentration and activity of SSAO and ALDH2 after CoQ10 administration. The results revealed that CoQ10 maintained the FA metabolic balance by preventing the elevation of SSAO expression (HH vs. HH + CoQ10, *p* = 0.0141) (Figure 3D), though exhibited no change in SSAO activity (HH vs. HH + CoQ10, *p* = 0.5990) (Figure 3E). Administration with CoQ10 could also prevent the decline of ALDH2 expression (HH vs. HH + CoQ10, *p* = 0.0054) (Figure 3F) and activity (HH vs. HH + CoQ10, *p* < 0.0001) (Figure 3G) under hypobaric hypoxic conditions. These findings indicated that CoQ10 can prevent FA accumulation not only by combination with FA,^14^, ^25^ but also by preserving its normal metabolism.

### 3.6 Nano‐packed coenzyme Q10 alleviated neurological deficits under acute hypobaric hypoxia via reducing formaldehyde accumulation

To investigate whether CoQ10 supplementation can prevent neurological deficits via FA elimination, we conducted the behavioral tests again. The results of open field test showed that the total distance (HH vs. HH + CoQ10, *p* = 0.0020) (Figure 3H), central distance (HH vs. HH + CoQ10, *p* = 0.0043) (Figure 3I), and the times of entries to the central zone (HH vs. HH + CoQ10, *p* = 0.0325) (Figure 3J) were rescued after CoQ10 administration. The results of novel objective recognition also showed that those treated with CoQ10 performed better in distinguishing the familiar objectives and the novel ones, compared to those non‐treated ones (HH vs. HH + CoQ10, *p* < 0.0001) (Figure 3K). Meanwhile, the results of step down test showed that the latency to electric shock was significantly increased (HH vs. HH + CoQ10, *p* = 0.0022) (Figure 3L), while the times of electric shock decreased (HH vs. HH + CoQ10, *p* = 0.0065) (Figure 3M) after CoQ10 supplementation. Besides, the neurological function of FA group was also significantly impaired, while improved after CoQ10 treatment, as exhibited by the HH group (Figure 3H–M). Furthermore, the neurological behavior of CTL was the same as the sham ones, indicating that CoQ10 exerted few side effects (Figure 3H–M). Taken together, these data suggested that pretreatment with CoQ10 can prevent neurological deficits probably by inhibiting FA accumulation in the brain.

### 3.7 Nano‐packed coenzyme Q10 prevented neuronal ferroptosis under acute hypobaric hypoxia via reducing formaldehyde accumulation

**FIGURE 4:**
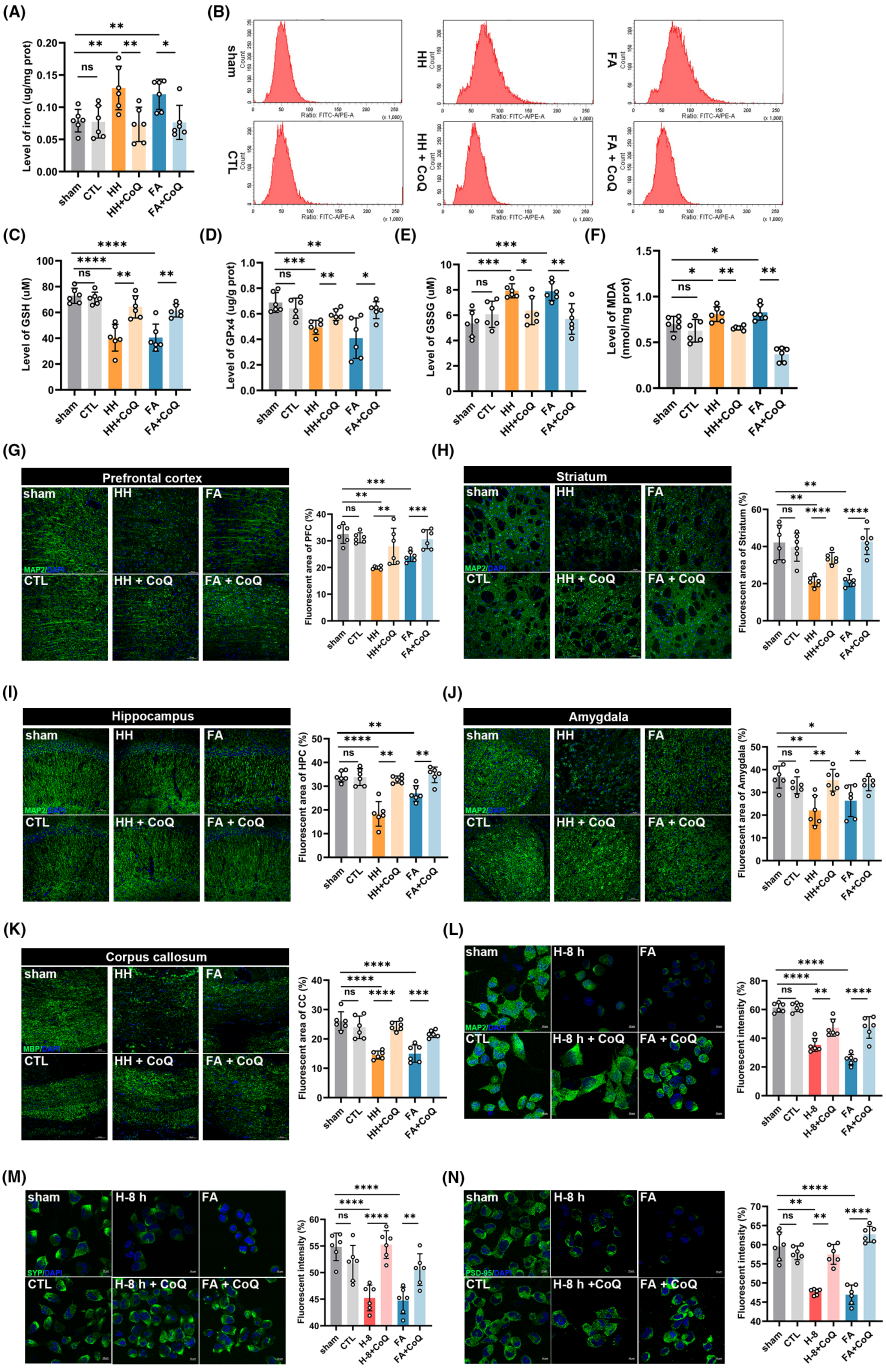
Nano‐packed coenzyme Q10 prevented neuronal ferroptosis under acute hypobaric hypoxia via reducing formaldehyde (FA) accumulation. (A) The levels of iron in the cerebrum detected with quantitative assay kit (HH vs. HH + CoQ10, *p* = 0.0093). (B) Lipid ROS detected by flow cytometry with a lipid peroxidation sensor (BODIPY 581/591 C11). The levels of cerebral GSH (HH vs. HH + CoQ10, *p* = 0.0015) (C), GPx4 (HH vs. HH + CoQ10, *p* = 0.0070) (D), GSSG (HH vs. HH + CoQ10, *p* = 0.0122) (E), and MDA (HH vs. HH + CoQ10, *p* = 0.0022) (F) quantified by assay kits. Changes of the neuronal injuries in prefrontal cortex (HH vs. HH + CoQ10, *p* = 0.0022) (G), striatum (HH vs. HH + CoQ10, *p* < 0.0001) (H), hippocampus (HH vs. HH + CoQ10: *p* = 0.0022) (I) and amygdala (HH vs. HH + CoQ10: *p* = 0.0024) (J) detected by confocal with MAP2 (green) using confocal imaging. Scale bar = 50 μm. (K) Changes of myelin in corpus callosum detected by MBP using confocal imaging (HH vs. HH + CoQ10, *p* < 0.0001). Scale bar = 50 μm. (L) Changes of neurons quantified by fluorescent intensity detected by MAP2 using confocal imaging (H‐8 h vs. H‐8 h + CoQ10, *p* = 0.0028). Scale bar = 20 μm. Changes of presynaptic protein (H‐8 h vs. H‐8 h + CoQ10, *p* < 0.0001) (M) and postsynaptic protein (H‐8 h vs. H‐8 h + CoQ10, *p* = 0.0022) (N) quantified by fluorescent intensity of SYP and PSD‐95 respectively. Scale bar = 20 μm. FA: formaldehyde; sham: unexposed control group; CTL: unexposed to HH but intragastrically administrated with CoQ10; HH: exposed to acute hypobaric hypoxia; HH + CoQ: intragastric administration with CoQ10 prior to HH exposure; FA + CoQ: intraperitoneal injection of FA and intragastric administration with CoQ10; GSH: glutathione; GSSG: oxidized glutathione; GPx4: glutathione peroxidase 4; MDA: malondialdehyde; H‐8 h: hypoxia for 8 h; H‐8 h + CoQ10: hypoxia for 8 h and incubated with CoQ10; PFC: prefrontal cortex; HPC: hippocampus; CC: corpus callosum; MBP: myelin basic protein; MAP2: microtubule‐associated protein 2; DAPI: 2‐(4‐Amidinophenyl)‐6‐indolecarbamidine dihydrochloride; SYP: synaptophysin; PSD‐95: postsynaptic density 95. *N* = 6 in each group, with three independent experiments. Data are presented as mean ± SD. ns: *p* > 0.05, *p < 0.05, ** *p* < 0.01, ***p < 0.001, ****p < 0.0001

To explore whether endogenously accumulated FA could induce neuronal ferroptosis in vivo, and the protective mechanism of CoQ10, we investigated the concentrations of indicators related to ferroptosis, including iron, LPO, GSH, GPx4, GSSG, and MDA. The results showed that the levels of iron were significantly elevated in the HH and FA group, while decreased after CoQ10 treatment (sham vs. HH, *p* = 0.0084; HH vs. HH + CoQ10, *p* = 0.0093) (Figure 4A). Meanwhile, the peaks of LPO in the HH and FA group were right shifted (indicating the LPO of the living cells), while left shifted (indicating a reduced state) after CoQ10 administration (Figure 4B). Consistently, the levels of the redox substances GSH (sham vs. HH, *p* < 0.0001; HH vs. HH + CoQ10, *p* = 0.0015) (Figure 4C) and GPx4 (sham vs. HH, *p* = 0.0006; HH vs. HH + CoQ10, *p* = 0.0070) (Figure 4D) were decreased in HH and FA group, and elevated after CoQ10 supplementation, while GSSG (sham vs. HH, *p* = 0.0003; HH vs. HH + CoQ10, *p* = 0.0122) (Figure 4E), and MDA (sham vs. HH, *p* = 0.0463; HH vs. HH + CoQ10, *p* = 0.0022) (Figure 4F) were just on the contrary. In addition, the results of neuronal ferroptosis also showed no significance between the sham and CTL groups, and the HH and FA groups (Figure 4A‐F). Taken together, these data suggested that FA accumulation induced by acute hypobaric hypoxia exposure promoted neuronal ferroptosis, and could be prevented by pretreatment with CoQ10.

To investigate the changes of the brain tissue and neurons after CoQ10 supplementation, we conducted immunofluorescent staining to reveal the injuries of neuron and myelin in vivo, as well as neuronal and synaptic alteration in vitro. Administration with CoQ10 prevented neuronal injuries in multiple brain regions, including prefrontal cortex (HH vs. HH + CoQ10, *p* = 0.0022) (Figure 4G), striatum (HH vs. HH + CoQ10, *p* < 0.0001) (Figure 4H), hippocampus (HH vs. HH + CoQ10, *p* = 0.0022) (Figure 4I), amygdala (HH vs. HH + CoQ10, *p* = 0.0024) (Figure 4J), as well as myelin loss in corpus callosum (HH vs. HH + CoQ10, *p* < 0.0001) (Figure 4K). Consistently, the in vitro experiments showed that incubation with CoQ10 can prevent neuronal (H‐8 h vs. H‐8 h + CoQ10, *p* = 0.0028) (Figure 4L), and synaptic loss [(SYP: H‐8 h vs. H‐8 h + CoQ10, *p* < 0.0001) (Figure 4M); (PSD‐95: H‐8 h vs. H‐8 h + CoQ10, *p* = 0.0022) (Figure 4N)] under hypoxia conditions. The neuronal and brain injuries in FA group were the same as the HH group, and the changes were also similar between the sham and the CTL groups (Figure 4G–N). These data indicated that CoQ10 prevented neuronal injuries and protected neurological function via inhibiting neuronal ferroptosis, with few side effects.

## 4 DISCUSSION

Our results demonstrated that acute hypobaric hypoxia exposure disturbed FA metabolism and induced FA accumulation in the neurons and the cerebrum, which further promoted neuronal ferroptosis and resulted in the neurological deficits for the first time. Pretreatment with CoQ10 could prevent endogenous FA accumulation and inhibit neuronal ferroptosis to preserve neurological function, which may be a potential preventative therapy for acute mountain sickness.

Acute hypobaric hypoxia exposure is a major challenge for visitors and sojourners at plateau,^26^ particularly the induced neurological dysfunction. In this study, the mice under simulated acute hypobaric hypoxia exposure also exhibited disturbed psychosis, and impaired cognition. Excessive FA exposure impaired learning and memory abilities,^27^ and eliminating FA alleviated neurological deficits.^14^ The FA concentration has been found increased in acute and chronic hypoxic conditions.^28^ It may be related to the activation of one‐carbon metabolism, and disturbed FA metabolism.^29^ In the current study, we observed that both the FA levels in the primary neurons and the cerebrum were significantly elevated. That is to say, excessive FA may act as a direct and critical role in neurological impairment under acute hypobaric hypoxia circumstances.

FA is mainly generated by methylamine deamination via SSAO,^7^ and eliminated by cytosolic alcohol dehydrogenase‐3 (ADH3) and mitochondrial ALDH2 in the neurons.^30^ ALDH2 is more critical in conditions with depleted GSH and accumulated FA,^8^, ^9^ because ADH3 is GSH‐dependent and has a lower K_M_‐value.^31^ The increased concentration of SSAO in our study was consistent with other acute hypoxic diseases.^10^ However, there was no significant change in the SSAO activity in the brain, which may relate to the location of SSAO and the duration of hypoxia. The activity of SSAO is different in diverse cell types after hypoxia,^32^, ^33^ which also shows a time‐dependent mode, as it increased in 6 h and decreased after 24 h hypoxia.^11^ Consistently, our cellular experiment showed that the activity of SSAO was gradually increased and peaked at 8 h. In addition, the hypothalamic–pituitary–adrenal (HPA) axis is activated in hypoxic conditions,^34^ and then triggered methylamine release and FA formation.^35^ On the other hand, the decreased activity and expression of ALDH2 observed in our study may be related to the down‐expression of von Hippel Lindau (VHL).^36^, ^37^ Moreover, the changes of SSAO and ALDH2 revealed in vitro was consistent with those of the acute high‐altitude sickness patients, whose symptoms are the most severe on the second day of hypoxia exposure.^38^ These evidences together proved that the alteration of SSAO and ALDH2 after acute hypobaric hypoxia exposure induced cerebral FA accumulation.

Ferroptosis is an iron‐dependent and LPO‐dependent form of regulated cell death.^39^ It is involved in multiple acute and chronic hypoxic neurological diseases.^40^, ^41^ In our study, the activation of the neuronal ferroptosis in the cerebrum after acute hypobaric hypoxia was observed and may be caused by the cerebral FA accumulation. Though the exact mechanisms needed further exploration, ALDH2 inhibition can directly promote ferroptosis in animal models.^42^ The concentrations of MDA (production of ferroptosis) and LPO were significantly decreased after ALDH2 activation,^43^ while increased in *ALDH2 KO* mice.^44^ Taken together, the disturbed FA metabolism and accumulated FA after acute hypobaric hypoxia exposure may evoke neuronal ferroptosis, and subsequent neurological dysfunction.

Excessive cerebral FA shows critically detrimental effect on the nervous system, while CoQ10 administration is useful in multiple neurological diseases.^45^ It can directly combine with the FA molecule for elimination and function preservation.^14^, ^25^ It can also inhibit ALDH2 inactivation to promote clearance.^46^ In this study, intragastric administration with CoQ10 for 3 days before acute hypobaric hypoxia exposure reduced neuronal FA accumulation, ferroptosis and brain injuries, with better behavioral outcomes, indicating that CoQ10 can prevent cognitive decline, at least partially, via FA elimination after acute hypobaric hypoxia. Importantly, neuronal and brain morphology, and neurological function of CoQ10‐treated group were the same as sham group, indicating no damage to the nervous system. Therefore, CoQ10 may be used as a potentially valuable drug for clinical prevention in high‐altitude exposure with few side effects.

There are some limitations in this study. First, gene editing or NaHSO_3_ (a specific scavenger, can damage cell membrane and axonal transport^47^) was not used to prove the exact role of FA in acute hypobaric hypoxia exposure mediated by the changes of SSAO and ALDH2. Second, although we proved the elimination of FA by CoQ10, we can not rule out other mechanisms that alleviate neurological impairment relating to CoQ10. Furthermore, the exact molecular mechanisms of neuronal ferroptosis caused by accumulated cerebral FA needs to be further studied. Apart from formaldehyde accumulation, other mechanisms such as blood brain barrier damage,^48^, ^49^ oxidative stress,^50^ excessive mitophagy and neuroinflammation,^51^ may also impair neurological function in hypoxia conditions and need to be further explored. Last, only male mice were used in our study. Many recent studies have reported the sex disparities in blood flow,^52^ brain metabolism,^53^ microvessels,^54^ and responses to hypoxia,^55^ thus the role of FA and protection of CoQ10 in female mice also need to be investigated in the future.

## 5 CONCLUSION

Acute hypobaric hypoxia exposure induced FA accumulation in the cerebrum and induced neuronal ferroptosis, resulting in neurological deficits. Pre‐administration with CoQ10 can avoid FA accumulation, inhibit neuronal ferroptosis, and maintain neurological functions, which may be a promising preventive strategy in clinic.

## CONFLICTS OF INTEREST

All authors declare no conflict of interests.

## AUTHORS’ CONTRIBUTIONS

XMJ and ZQT designed and supervised the study. XYW, HCS, and XW performed the experiments and collected the data. XYW drafted and finalized the manuscript. LLC and CHR edited the manuscript. All authors read and approved the final manuscript.

## Supporting information

Fig S1

Fig S2

Fig S3

## Data Availability

The datasets used and/or analyzed during the current study are available from the corresponding author upon reasonable request.
