# Supplementary figures and images for: Acute high‐altitude hypoxia exposure causes neurological deficits via formaldehyde accumulation
Source: CNS Neurosci Ther. 2022 May 18;28(8):1183–94. doi: 10.1111/cns.13849 (PMC9253739; doi:10.1111/cns.13849)

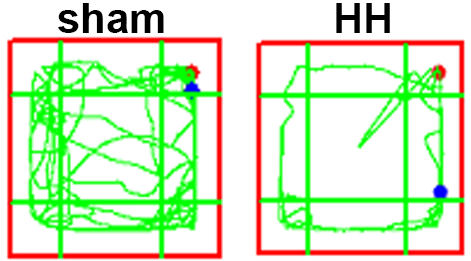

Supplement: Supplementary file 1 — Fig S1 [file CNS-28-1183-s003.tiff]

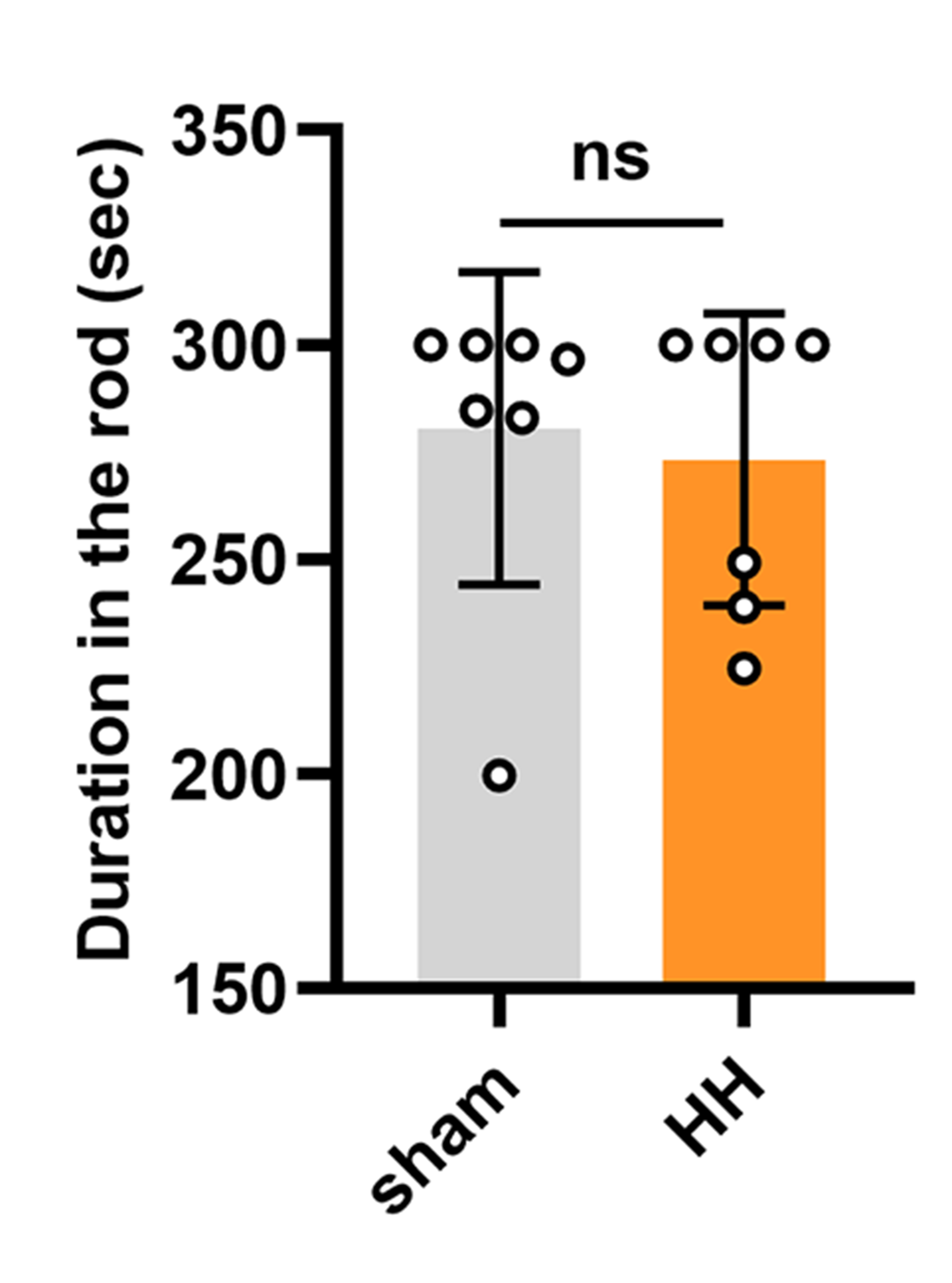

Supplement: Supplementary file 2 — Fig S2 [file CNS-28-1183-s001.tiff]

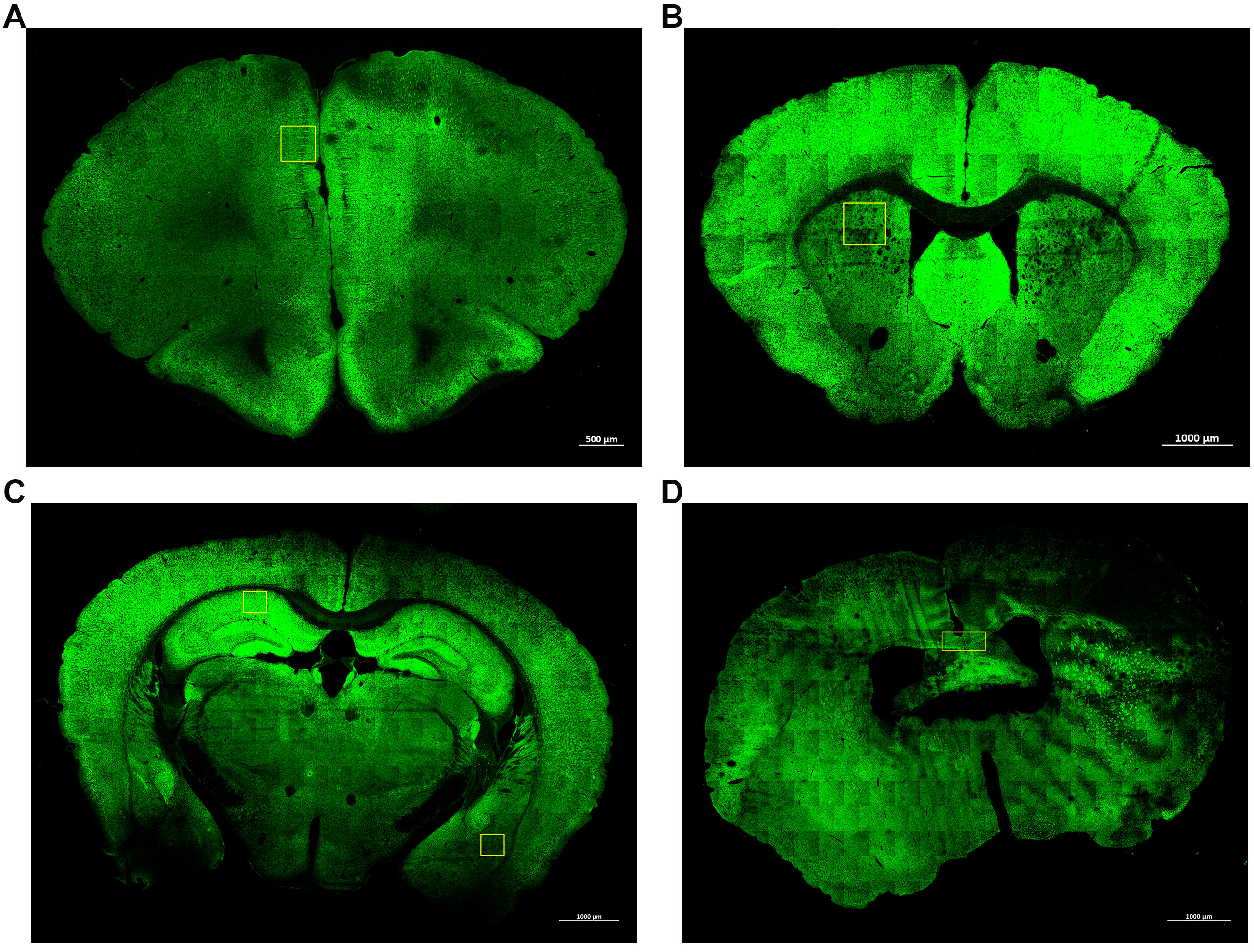

Supplement: Supplementary file 3 — Fig S3 [file CNS-28-1183-s002.tiff]
